# Functioning and quality of life in patients with neuropathy associated with anti-MAG antibodies

**DOI:** 10.1007/s00415-018-9081-7

**Published:** 2018-10-10

**Authors:** Yuri M. Falzone, Marta Campagnolo, Mariangela Bianco, Patrizia Dacci, Daniele Martinelli, Marta Ruiz, Silvia Bocci, Federica Cerri, Angelo Quattrini, Giancarlo Comi, Luana Benedetti, Fabio Giannini, Giuseppe Lauria, Eduardo Nobile-Orazio, Chiara Briani, Raffaella Fazio, Nilo Riva

**Affiliations:** 1 Division of Neuroscience, Department of Neurology, Institute of Experimental Neurology (INSPE), IRCCS San Raffaele Scientific Institute, Milan, Italy; 2 Department of Neurosciences, Sciences University of Padova, Padova, Italy; 3 Neuromuscular and Neuroimmunology Service, Department of Medical Biotechnology and Translational Medicine, IRCCS Humanitas Clinical and Research Institute, Milan University, Rozzano, Milan Italy; 4 Neurologia III, Fondazione IRCCS Istituto Neurologico “Carlo Besta”, Milan, Italy; 5 Department of Biomedical and Clinical Sciences “Luigi Sacco”, University of Milan, Milan, Italy; 6 Università Vita e Salute San Raffaele, Milan, Italy; 7 Department of Neuroscience, Rehabilitation, Ophthalmology, Genetics, Maternal and Child Health, University of Genova, IRCCS Policlinico San Martino, Genova, Italy; 8 Department of Medical, Surgical and Neurological Sciences, Neurology and Clinical Neurophysiology Unit, University of Siena, Siena, Italy

**Keywords:** Chronic inflammatory polyneuropathy, Pain, MGUS, Rehabilitation, Walking ability, Balance

## Abstract

Although anti-myelin-associated glycoprotein (MAG) antibody neuropathy is reported as a slowly progressive disease, it can lead to significant disability and impairment of health-related quality of life (HR-QoL) and social participation. The aim of this cross-sectional study was to evaluate the functioning and HR-QoL determinants in 67 patients with anti-MAG neuropathy in terms of the International Classification of Functioning, Disability, and Health (ICF). Evaluations included: Medical Research Council (MRC) sum score, Sensory Modality Sum score (SMS), Berg balance scale (BBS), Fatigue Severity Scale (FSS), Visual Analogue Scale (VAS) for pain, 9-Hole Peg Test (9-HPT), 6-min Walk Distance (6MWD), Impact on Participation and Autonomy (IPA) and the physical component score (PCS) and mental component score (MCS) of the short-form-36 health status scale (SF-36) HR-QoL measure. In the regression models, 6MWD was the most reliable predictor of PCS, explaining the 52% of its variance, while the strongest determinants of 6MWD were BBS and FSS, explaining the 41% of its variance. Consistently, VAS and BBS were good predictor of PCS, explaining together 54% of its variance. FSS was the most reliable determinant of MCS, explaining 25% of its variance. SMS and MRC were not QoL determinants. The results of our study suggest that 6MWD and FSS might be considered as potential meaningful outcome measures in future clinical trials. Furthermore, neurorehabilitation interventions aimed at improving balance and walking performance, fatigue management, and specific pain relief therapy should be considered to ameliorate participation in social life and HR-QoL in anti-MAG neuropathy patients.

**Electronic supplementary material:**

The online version of this article (10.1007/s00415-018-9081-7) contains supplementary material, which is available to authorized users.

## Introduction

Anti-myelin-associated glycoprotein (MAG) neuropathy is a distal symmetric, predominantly sensory polyneuropathy [1–3] associated with monoclonal immunoglobulin M (IgM) reactive towards MAG [2, 4–6]. Although anti-MAG neuropathy is usually slowly progressive, it can sometimes lead to persistent disability and reduced autonomy [2, 3, 7, 8]. Unfortunately, evidence-based treatment strategies are still lacking [9]. The International Classification of Functioning, Disability, and Health (ICF) of the World Health Organization (WHO), has provided researchers with a framework to understand the interacting consequences of any health condition, ranging from impairments (deficits in body structures and functions) to activity limitations, and participation restrictions [10]. According to the ICF, functioning embraces the role of all body functions (e.g., sensory function), activities (e.g., walking ability), and social participation, including personal (e.g., gender, age) as well as environmental factors (e.g., working place). Therefore, the aim of this study was to evaluate patient’s functioning and health-related quality-of-life (HR-QoL) determinants in terms of the ICF classification, to identify meaningful outcome measure to be used in clinical trials and potential targets for tailored interventions in patients with anti-MAG neuropathy.

## Patients and methods

### Subjects and study design

We did this investigator-initiated, multicentric cross-sectional study in six tertiary referral hospital, located in the northern and central Italy, all with a special expertise and interest in peripheral nervous system (PNS) disorders. The study was approved by the research ethics committees of each participating center. Inclusion criteria were: patients aged between 18 and 85 years; anti-MAG neuropathy established according to European Federation of Neurological Societies (EFNS) and PNS diagnostic criteria [2]; presence of IgM monoclonal gammopathy of undetermined significance (MGUS) [11] or Waldenström’s macroglobulinemia [12]; positive serum anti-MAG antibodies (cut-off positive value on ELISA > 1000 Buhlmann titer unit, BTU) [13]; neurophysiological tests consistent with a distal sensory-predominant demyelinating neuropathy [2, 14]. Patients presenting with any other neurological, musculoskeletal, or medical disorders expected to influence outcome-measure scoring were excluded. Patients were recruited regardless of previously administered immunomodulatory therapies.

### Assessment

Clinical and demographic data were recorded for each patient. Evaluation of patient’s functioning and health-related quality-of-life (HR-QoL) determinants was performed in terms of the ICF classification. Assessment of body functions included: muscle strength, evaluated with the Medical Research Council (MRC) sum score (12 muscle for each side) [15, 16]; sensory function, rated according to the Sensory Modality Sum score (SMS), as described [17]; balance, assessed with Berg Balance Scale (BBS) [17, 18]; fatigue, assessed with the self-reported Rasch built 7 item modified Fatigue Severity Scale (FSS) [19] and pain, assessed with the Visual Analogue Scales (VAS**)** [20, 21]. Dexterity, rated with the 9-hole peg test (9-HPT) [22, 23], and walking performance, evaluated with the 6-min walk distance (6MWD) [24, 25], were considered as reliable activity measures for upper and lower limbs, respectively. The 9-HPT time score was calculated by averaging three attempts in the dominant hand. Social participation was evaluated with the self-assessment questionnaire the Impact on Participation and Autonomy (IPA) [26–28]. Two different subscales, the autonomy indoors (IPA indoors) and autonomy outdoors (IPA outdoors) were considered [17, 29]. Health-related quality of life (HR-QoL) was assessed using self-reported Medical Outcome Study 36-item short-form health status scale (SF-36) [30]. The 36 items were aggregated to score the eight scales in turn to calculate the physical component summary score (PCS) and the mental component summary score (MCS) [16, 30, 31]. The patient’s evaluation was performed, after informed consent, by a single examiner, who had more than 5 years of clinical experience with neuropathic patients.

### Statistical analyses

A sample size of 65 was required to achieve 95% of power to detect an R2 of 25% attributed to a maximum of five independent variables model using an *F* test (with a significance level *α* = 0.05). Patients characteristics were analyzed using descriptive statistics, quantitative data are given in mean and standard deviation (SD). Correlation analyses were performed among determinants in two consecutive steps. Initially, correlations (Pearson’s *r*) among demographic features, body functions, activities, participation and quality-of-life outcome measures were performed. Correlation outcome scores were interpreted as follows: very weak (*r* ≤ 0.20); weak (0.20 > *r* ≤ 0.40); moderate (0.40 > *r* ≤ 0.70); strong (0.70 > *r* ≤ 0.90); very strong (*r* > 0.90) as described [32]. Subsequently hierarchical multiple univariate linear regression analysis (stepwise procedure) was then carried out to underline: (1) which body functions (sensory function, muscle strength, balance, pain and fatigue) best explained variance in activities, participation and HR-QoL measures, respectively; (2) which activities (dexterity and walking ability) best explained the variance in participation and HR-QoL; (3) which participation scores (autonomy indoors and outdoors) best explained the variance in HR-QoL. The results were adjusted for age. The strength of the association between the dependent variable and the independent variables was expressed as a percentage (adjusted R2 × 100), and the relative importance of the independent variables was given as a standardized coefficient β. *P* values < 0.05 were considered significant. The Statistical Package for Social Sciences (SPSS version 22.0) was used to perform the analyses.

## Results

Sixty-seven patients (24 women, 43 men), with mean age at neuropathy onset 69.2 years (SD 8.0) and mean disease duration 7.2 years (SD 4.8), were recruited. Functional outcome in terms of ICF classification and HR-QoL measures are shown in Table 1. Our population presented with a predominantly sensory neuropathy with balance impairment, indeed SMS score was altered in almost all patients (65/67, 97.0%), and BBS score in 56 patients (83.6%). Considering sensory impairment, the most affected SMS sub-score was vibration sense, which was abnormal in 56 patients (83.6%), followed by position sense altered in 44 patients (65.7%) (Online Resource 1). Fatigue and pain were reported by 57 (85.1%) and 49 patients (73.1%), respectively, while MRC score was slightly impaired in 43 patients (64.2%).

**Table 1:** Functional outcome and HR-QoL of 67 patients with anti-MAG neuropathy

Variable	Mean score (SD)
Arm functioning	
MRC arms	67.5 (5.4)
SMS arms	26.2 (3.4)
VAS arms	1.3 (2.2)
FSS	9.0 (6.8)
Leg functioning	
MRC legs	45.6 (6.6)
SMS legs	19.2 (5.8)
VAS legs	3.1 (2.8)
FSS	9.0 (6.8)
BBS	47.6 (8.1)
Activities	
9-HPT (s)	30.8 (14.0)
6MWD (m)	356.0 (140.8)
Participation	
IPAI	0.8 (0.8)
IPAO	1.3 (1.1)
Quality of life	
PCS	40.9 (10.3)
MCS	46.2 (10.5)

Values are mean; *SD* standard deviation, *MRC arms* medical research council sum score upper limbs (range 0–70, higher values indicate better muscle strength), *MRC legs* medical research council sum score lower limbs (range 0–50, higher values indicate better muscle strength), *SMS arms* sensory modality sum score upper limbs (range 0–28, higher values indicate better sensory function), *SMS legs* sensory modality sum score lower limbs (range 0–28, higher values indicate better sensory function), *VAS arms* visual analogue scale upper limbs (range 0–10, lower values indicate less pain intensity), *VAS legs* visual analogue scale lower limbs (range 0–10, lower values indicate less pain intensity), *FSS* 7-item Rasch built Fatigue Severity Scale (range 0–21, lower values indicate less fatigue), *BBS* Berg balance scale (range 0–56, higher scores indicate better balance performance), *9-HPT* 9-hole peg test (time score average of three attempts in dominant hand, higher time indicates lower dexterity performance), *6MWD* 6 min walking distance (maximum 600 m, higher values indicate better walking performance), *IPAI* impact on participation and autonomy indoors (range 0–4, lower values indicate better autonomy), *IPAO* impact on participation and autonomy outdoors (range 0–4, lower values indicate better autonomy), *MCS* mental component summary (range 0–100, higher scores indicate better health), *PCS* physical component summary (range 0–100, higher scores indicate better health)

The results of the correlations studies and hierarchical multiple univariate linear regression analysis with stepwise strategy are shown in Tables 2 and 3, respectively. Of note, the activity measure 9-HPT correlated with MRC score (*r* = − 0.59, *p* < 0.01) and SMS arms score (*r* = − 0.52, *p* < 0.01), while 6MWD correlated with BBS (*r* = 0.55, *p* < 0.01) and FSS score (*r* = − 0.51, *p* < 0.01). The social participation measure IPAO correlated with BBS (*r* = − 0.61, *p* < 0.01) and FSS (*r* = 0.61, *p* < 0.01), while IPAI correlated with SMS (*r* = − 0.62, *p* < 0.01) and BBS (*r* = − 0.61, *p* < 0.01). Concerning HR-QoL, PCS correlated with 6MWD (*r* = 0.74, *p* < 0.01) and with IPAO (*r* = − 0.72, *p* < 0.01), while MCS moderately correlated with body functions such as FSS (*r* = − 0.45, *p* < 0.01) and with participation measure IPAI (*r* = − 0.44, *p* < 0.01) and IPAO (*r* = − 0.42, *p* < 0.01). The strength of the correlations between PCS with 6MWD and IPAO was high, while it was moderate for all the other correlations.

**Table 2:** Pearson correlation of variables related to demographic features, functioning, participation and physical and mental status scores

	9HPT	6MWD	IPAI	IPAO	PCS	MCS
Age	0.09	− 0.30	0.22	0.17	− 0.16	− 0.01
MRC@	− 0.59**	0.39**	− 0.50**	− 0.52**	0.34**	0.29*
SMS@	− 0.52**	0.37**	− 0.62**	− 0.54**	0.44**	0.18
BBS		0.55**	− 0.61**	− 0.61**	0.55**	0.15
FSS	0.12	− 0.51**	0.54**	0.61**	− 0.46**	− 0.45**
VAS	0.22	0.20	0.44**	0.55^**^	− 0.53**	− 0.26*
9HPT			0.48**	0.43**	− 0.33**	− 0.25
6MWD			− 0.57**	− 0.57**	0.74**	0.20
IPAI					− 0.62**	− 0.44**
IPAO					− 0.72**	− 0.42**

*MRC* medical research council sum scores, *SMS* sensory modality sum score, *VAS* visual analogue scale, *FSS* fatigue severity scale, *9-HPT* 9-hole peg test, *6MWD* 6-min walking distance, *IPAI* impact on participation and autonomy indoors, *IPAO* impact on participation and autonomy outdoors, *MCS* mental component summary, *PCS* physical component summary

*p < 0.05; **p < 0.01; @MRC and SMS score were divided in upper and lower limbs subscales when correlated with activity measures

**Table 3:** Hierarchical multiple univariate linear regression analysis (stepwise procedure) of associations between body functions, activities, participation and quality of life

Predictor variable, and regressed variable	*β* weight	Multiple *r*^2^ model × 100	Test
Body function			
MRC arms	− 0.43**	0.47	9HPT
SMS arms	− 0.37**		
BBS	0.42**	0.41	6MWD
FSS	− 0.39**		
SMS	− 0.49**	0.57	IPAI
BBS	− 0.36**		
FSS	0.19*		
FSS	0.39**	0.64	IPAO
VAS	0.28**		
SMS	− 0.26*		
BBS	− 0.24*		
VAS	− 0.53**	0.54	PCS
BBS	0.37*		
FSS	− 0.46**	0.25	MCS
Activity			
6MWD	− 0.45**	0.39	IPAI
9-HPT	0.33**		
6MWD	− 0.46**	0.38	IPAO
9-HPT	0.30**		
6MWD	0.73**	0.52	PCS
6MWD	0.26*	0.05	MCS
Participation			
IPAO	− 0.73**	0.52	PCS
IPAO	− 0.44**	0.18	MCS

Strikethrough variables indicate no independent correlation

*MRC arms* medical research council sum score upper limbs, *MRC legs* medical research council lower limbs, *SMS arms* sensory modality sum score upper limbs, *SMS legs* sensory modality sum score lower limbs, *VAS arms* visual analogue scale upper limbs, *VAS legs* visual analogue scale upper limbs, *FSS* fatigue severity scale, *9-HPT* 9-hole peg test, *6MWD* 6 min walking distance, *IPAI* impact on participation and autonomy indoors, *IPAO* impact on participation and autonomy outdoors, *MCS* mental component summary, *PCS* physical component summary

*Significance of β weight *p* < 0.05

**Significance of *β* weight *p* < 0.01

Considering hierarchical multiple univariate linear regression analysis (Table 3; Online Resource 1), MRC arms and SMS arms scores were independently associated with the dexterity measure 9-HPT, explaining 47% of its variance, being upper limbs muscle strength the main determinant based on *β*-values (*β* = − 0.43). The 41% of the total variance of the activity measure 6MWD was explained by BBS (main determinant; *β* = 0.42) and FSS. Regarding social participation, the 57% of the whole variance of IPAI was explained by the body functions SMS, BBS and FSS, being sensory function the main determinant (*β* = − 0.49). The 64% variance of IPAO was explained by FSS, VAS, SMS and BBS; considering β-values fatigue was the main determinant (*β* = 0.39). The 54% of the total variance of the HR-QoL measure PCS was explained by pain VAS (*β* = − 0.53, main determinant) and BBS (*β* = 0.37); while 25% of the MCS variance was explained by FSS (*β* = − 0.46). According to *β* values, 6MWD was the main activity measure independently associated with IPAO, IPAI, MCS and PCS, while IPAO was the only participation measure independently associated with both QoL measures.

## Discussion

In this multicentre, cross-sectional study, we observed that sensory function, balance, fatigue and walking ability were the strongest determinants of patient’s participation in social life. On the other hand, walking ability, balance, pain and limitation in outdoor autonomy were significant predictors of physical aspects of QoL, while fatigue was the only determinant of mental aspects of QoL.

Balance, as measured with BBS, was the main determinant of walking ability, highlighting the relevance of gait ataxia in anti-MAG neuropathy patients consistent with the well-known clinical features [3]. In addition, balance was a significant predictor of social participation and physical QoL. Therefore, neurorehabilitation focused on balance exercise should be considered to ameliorate the walking performance and subsequently the autonomy and QoL perception. Furthermore, BBS might be evaluated as potential candidate outcome measure in future clinical trials, even if more confirmatory investigations are needed.

We observed that sensory impairment was the main determinant of participation, suggesting that sensory disturbances markedly interfere with patient’s autonomy. Consistently, almost whole population presented with sensory impairment, namely vibration and position sense, predominantly at the lower limbs. However, sensory function was not independently correlated with QoL disfavoring its use as outcome measure in clinical trials [33]. Similarly, muscle strength did not show any independent correlation with social participation and QoL, which is unsurprising in such a mainly sensory disorder.

Fatigue and pain were symptoms reported by the majority of our patients. Of note, fatigue was the only predictor of mental QoL, explaining approximately 25% of its variance, while pain was the strongest determinant of physical QoL among body functions, in keeping with the results from recent reports highlighting the relevance of pain as well as cramps in anti-MAG neuropathy [33, 34]. These data suggest that appropriate algological and fatigue treatments should be considered to improve mental and physical QoL perception in patients with anti-MAG neuropathy. Furthermore, fatigue might be considered as a proper candidate outcome measure in future clinical trials. Walking performance resulted lower than reference values in healthy subjects [35]. Of note, 6MWD was the strongest determinant of physical QoL, explaining independently approximately half of its variance. Furthermore, it was a significant predictor of participation and mental QoL. Conversely, in a previous study, walking ability as measured with timed 10 m walk (10 MWT), did not result as a significant determinant of QoL [8]. Hence, 6MWD compared with ten MWT might be a more reliable outcome measure in future clinical trials and suitable for the evaluation of walking performance in patients with anti-MAG neuropathy. Furthermore, physical therapy focused on walking ability might be considered to improve participation and QoL perception.

We noticed that limitation in outdoor autonomy had a major impact on general QoL with no effect observed for indoor autonomy. Therefore, in anti-MAG neuropathy patients, limitations in social contacts, leisure, and mobility outdoors have a higher impact on QoL than self-care and mobility indoors.

This is the first study demonstrating that QoL, measured with SF36 questionnaire, was lower in patients with anti-MAG antibody neuropathy compared with the Italian general population [7], consistently with a previous study performed in French and English anti-MAG neuropathy population [8]. Furthermore, the physical aspects were more impaired than mental aspects of QoL, supporting the relevance of physical signs and symptoms to overall QoL in neuropathic patients.

This is the first study detailing the functioning of patients with anti-MAG neuropathy in terms of the ICF classification. However, we acknowledge that this study has also limitations. The current analysis did not include a tremor score, which is one of the most relevant symptoms of anti-MAG neuropathy. However, although tremor could theoretically have influenced patients’ dexterity, our study showed that the dexterity measure 9-HPT was not a relevant determinant of neither participation nor HR-QOL. Moreover, in the Delmont study the tremor score, although correlated ad univariate analysis, did not prove to be independently associated with the SF-36 QoL sub-score PCS, indirectly confirming the interpretation of our results [33]. A further limit is intrinsic to our study design, which, consistently with the ICF classification, included social participation but not social support. This limit is complemented by the Delmont study which, although lacking an evaluation of social participation, did include a survey assessing social support showing a good correlation with PCS, even if not confirmed by multivariate analysis. Finally, not all the instruments we used were psychometrically validated in anti-MAG patients, even though they are widely employed in the neurorehabilitation field [16, 17].

Although randomized clinical trials regarding anti-MAG neuropathy failed to overcome primary outcome measures [9, 36, 37], some immunotherapies may be effective if they were administered before the progression of axonal damage [38]. Hence, sensitive outcome measures may reveal the efficacy of treatment in appropriately selected patients. The indices evaluated in this study might, therefore, be considered as outcome measures in future clinical trials. In particular, our data show that that walking ability was the most reliable predictor of physical aspect of QoL, while fatigue was the only determinant of mental aspect of QoL in patients with anti-MAG neuropathy. Similarly, walking performance and fatigue correlated well with participation measures. Finally, considering that evidence-based treatment strategies are lacking in anti-MAG neuropathy, neurorehabilitation aimed at improving balance and walking performance, fatigue management, and specific pain relief therapy should be considered to ameliorate social participation and QoL perception in these patients.

## Electronic supplementary material

Below is the link to the electronic supplementary material.

Supplementary material 1 (PDF 345 KB)
