# Supplementary material for: Functioning and quality of life in patients with neuropathy associated with anti-MAG antibodies
Source: J Neurol. 2018 Oct 10;265(12):2927–33. doi: 10.1007/s00415-018-9081-7 (PMC6244677; doi:10.1007/s00415-018-9081-7)
Supplement: Supplementary file 1 — Supplementary material 1 (PDF 345 KB) [file 415_2018_9081_MOESM1_ESM.pdf]

Functioning and quality of life in patients with neuropathy associated with anti-MAG antibodies.

Yuri Matteo Falzone,[1] Marta Campagnolo,[2] Mariangela Bianco,[3] Patrizia Dacci,[4] Daniele Martinelli,[1] Marta Ruiz[2], Silvia Bocci,[8] Federica Cerri,[1] Angelo Quattrini,[1] Giancarlo Comi,[1,6] Luana Benedetti,[7] Fabio Giannini,[8] Giuseppe Lauria,[4,5] Eduardo Nobile-Orazio,[3] Chiara Briani,[2] Raffaella Fazio,[1,a] Nilo Riva,[1,a]

[1] Department of Neurology, Institute of Experimental Neurology (INSPE), Division of Neuroscience, IRCCS San Raffaele Scientific Institute, Milan, Italy

[2] Department of Neurosciences, Sciences University of Padova, Padova, Italy.

[3] Neuromuscular and Neuroimmunology Service, Department of Medical Biotechnology and Translational Medicine, Milan University, IRCCS Humanitas Clinical and Research Institue, Rozzano, Milan.

[4] Neuroalgology Disease, IRCCS Foundation, "Carlo Besta" Neurological Institute, Milan, Italy.

[5] Department of Biomedical and Clinical Sciences "Luigi Sacco", University of Milan, Milan, Italy

[6] Università Vita e Salute San Raffaele, Milan, Italy

[7] Department of Neuroscience, Rehabilitation, Ophthalmology, Genetics, Maternal and Child Health, University of Genova, IRCCS Policlinico San Martino, Genova.

[8]Department of Medical, Surgical and Neurological Sciences, Neurology and Clinical Neurophysiology Unit, University of Siena, Italy

[a] These authors share senior authorship

Corresponding author: Nilo Riva, MD, PhD

Department of Neurology, Institute of Experimental Neurology (INSPE), Division of Neuroscience, IRCCS San Raffaele Scientific Institute, Milan, Italy

Corresponding author's phone and fax: + 39 -02 2643 2990

Corresponding author's e-mail address: riva.nilo@hsr.it

Word count: 2046

Fig S1. Hierarchical multiple univariate linear regression analysis (stepwise procedure) between body functions, activities, participation and physical component summary score (PCS) after adjustment for age

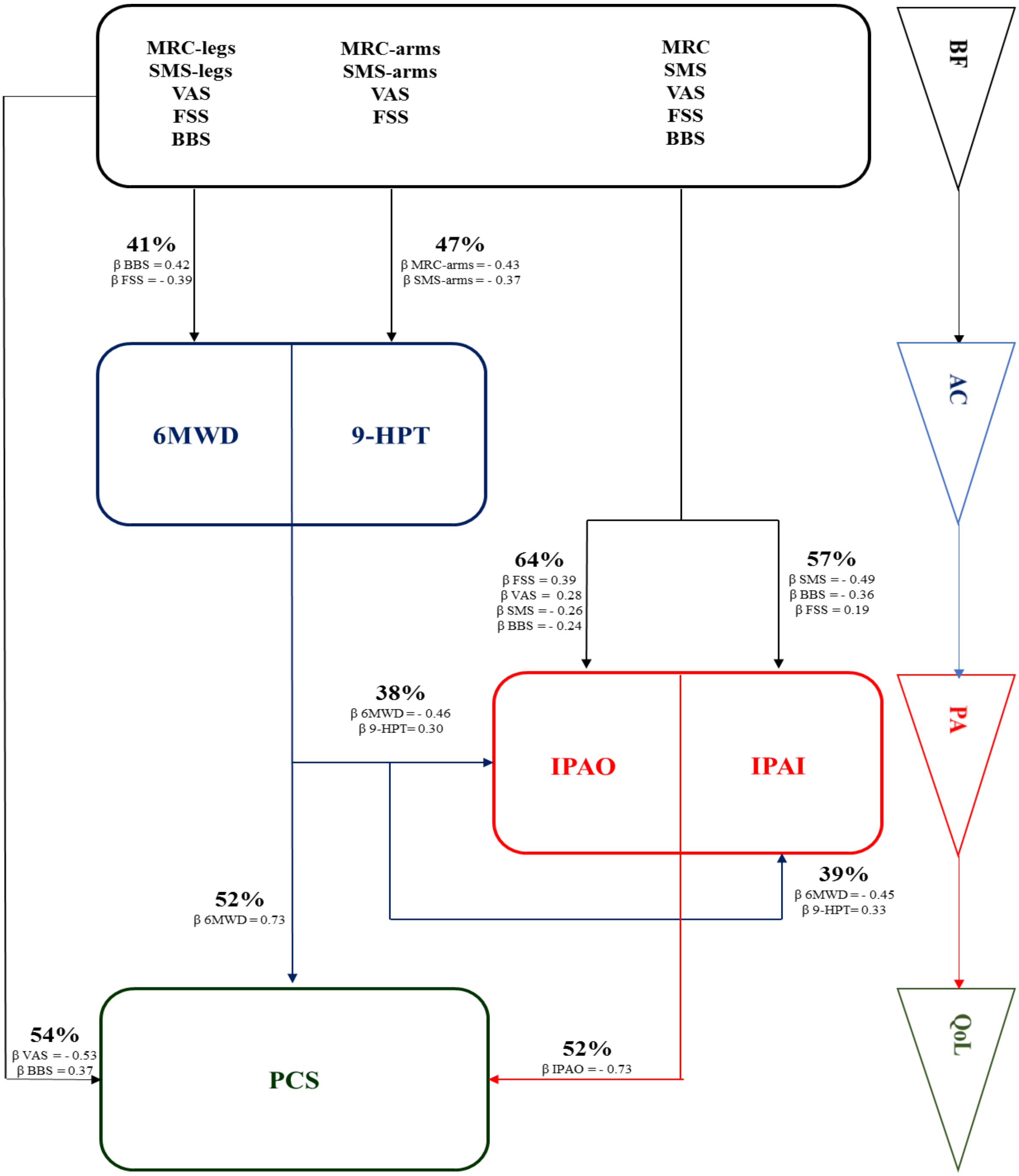

Interpretation (box 6MWD): the effect of independent body functions (represented by legs muscle strength, legs sensory function, pain, fatigue and balance) on dependent activity (example walking performance assessed with 6MWD). In this case, BBS and FSS resulted in a model which explained a total of 41% (Adjusted R2 · 100) of the total variance in walking performance scores, when adjusted for age. The relative contribution of the independent variables is expressed as β. MRC-arms, Medical Research Council sum score upper limbs; MRC-legs, Medical Research Council sum score lower limbs; SMS-arms, Sensory Modality Sum score upper limbs; SMS-legs, Sensory Modality Sum score lower limbs; VAS-arms, visual analogue scale; FSS, 7-item Rasch built Fatigue Severity Scale. BBS, Berg balance scale; 9-HPT, 9-hole peg test; 6MWD, 6 minute walking distance; IPAI, impact on participation and autonomy indoors; IPAO, impact on participation and autonomy outdoors; PCS, physical component summary. BF, body functions; AC, activities; PA, participation; QOL, quality of life.

Fig S2. Hierarchical multiple univariate linear regression analysis (stepwise procedure) between body functions, activities, participation and mental component summary score (MCS) after adjustment for age

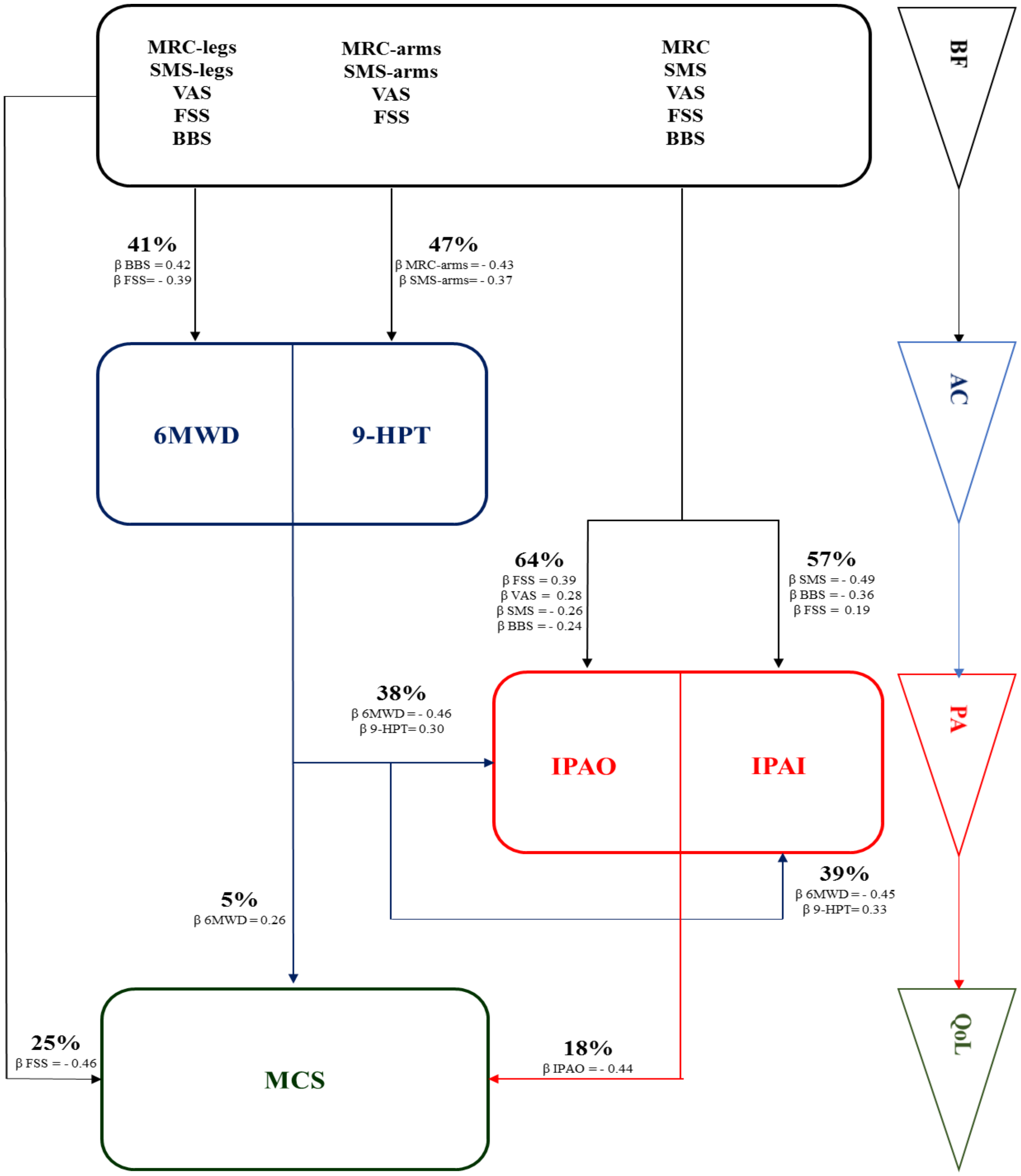

Interpretation (box MCS): the effect of independent activities (represented by walking performance and dexterity) on dependent MCS. In this case, 6MWD resulted in a model which explained a total of 5% (Adjusted R2 · 100) of the total variance in MCS, when adjusted for age. The contribution of the independent variable is expressed as β. MRC-arms, medical research council sum score upper limbs; MRC-legs, medical research council sum score lower limbs; SMS-arms, sensory modality sum score upper limbs; SMS-legs, sensory modality sum score lower limbs; VAS-arms, visual analogue scale; FSS, 7-item Rasch built Fatigue Severity Scale. BBS, Berg balance scale; 9-HPT, 9-hole peg test; 6MWD, 6 minute walking distance; IPAI, impact on participation and autonomy indoors; IPAO, impact on participation and autonomy outdoors; MCS, mental component summary. BF, body functions; AC, activities; PA, participation; QOL, quality of life.

Table S1. Sensory modality sum score (SMS) of 67 patients with anti-MAG neuropathy

Sensory Modality Sum score (SMS)		
**Vibration Sense (0-16 PTS)**	Mean score (SD)	12.2 (3.5)
	Impaired in upper limbs	22.2%
	Impaired in lower limbs	83.6%
**Position Sense (0-8 PTS)**	Mean score (SD)	6.0 (1.9)
	Impaired in upper limbs	5.5%
	Impaired in Mean score	65.7%
**Light touch sensation (0-16 PTS)**	Mean score (SD)	13.5 (2.6)
	Impaired in upper limbs	11.1%
	Impaired in lower limbs	55.5%
**Pin prick sensation (0-16 PTS)**	Mean score (SD)	14.9 (2.2)
	Impaired in upper limbs	5.9%
	Impaired in lower limbs	22.2%

SD, standard deviation; PTS, points; higher score indicate better sensory function.
